# P-1784. Analytical Validation of a Metagenomic Next Generation Sequencing (mNGS) Test for Agnostic Detection of Microbial Organisms in CNS Infections

**DOI:** 10.1093/ofid/ofaf695.1953

**Published:** 2026-01-11

**Authors:** Coren A Milbury, Brian O’Donovan, Emily Crawford, Nick Gimbrone, Sirisha Thippabhotla, Greg Gydush, Fay Wang, Anna Uehara, Timothy M Blicharz, Benisa Yuzawa, Peter Trefry, Wendy Brodeur, Tim DeSmet, Steve Miller

**Affiliations:** Delve Bio, Inc., Boston, Massachusetts; Delve Bio, Boston, Massachusetts; Delve Bio, Boston, Massachusetts; Delve Bio, Boston, Massachusetts; Delve Bio, Boston, Massachusetts; Delve Bio, Boston, Massachusetts; Delve Bio, Boston, Massachusetts; Delve Bio, Boston, Massachusetts; Delve Bio, Boston, Massachusetts; Delve Bio, Boston, Massachusetts; Broad Clinical Labs, Burlington, Massachusetts; Broad Clinical Labs, Burlington, Massachusetts; Broad Clinical Labs, Burlington, Massachusetts; Delve Bio, Boston, Massachusetts

## Abstract

**Background:**

Metagenomic next-generation sequencing (mNGS) enables simultaneous detection of viruses, bacteria, fungi and parasites from a single sample. Delve Bio, in collaboration with Broad Clinical Labs, has developed and validated a LDT to enable widespread access to agnostic and comprehensive pathogen detection in cerebrospinal fluid (CSF). The test, Delve Detect, utilizes molecular methods and automated lab processes for DNA and RNA extraction, library preparation and sequencing. Integration of internal and external controls ensures accuracy throughout lab processing and reporting. Proprietary bioinformatic analysis and a custom clinical interpretation interface have been separately validated.

**Methods:**

Validation of Delve Detect was performed using previously characterized de-identified clinical CSF samples with clinically relevant pathogens for evaluation of test accuracy. Limits of detection, limits of blank, threshold confirmation, repeatability, reproducibility, interference, specificity and cross-reactivity were all evaluated. Custom contrived sample matrices consisting of human content and clinically-relevant microbes were developed. Commutability was validated across 7 representative microbes by demonstrating equivalent limit of detection (LoD) values within the 95% CI compared to LoD in the human sample matrix.

**Results:**

Observed LoDs ranged from < 1 to 497 units/mL across organisms. Organism detection thresholds were confirmed and Limit of Blank exhibited a < 3% false positive detection rate. Intra- and inter-run repeatability and reproducibility were each 100% for all microbes at ∼1 log above LoD. Species-level specificity was evaluated in a cross-reactivity study of genetically similar species mixed at 5 titration levels. Diagnostic accuracy was demonstrated in 134 clinical samples (PPV 86.1%, NPV 97.5%). Endogenous and exogenous interfering substances were evaluated at multiple concentrations to understand potential impact of reagent carryover, hemolysis, and host background upon assay performance.

**Conclusion:**

Results demonstrate analytical validity of mNGS as a sensitive and specific test of pathogens. Integration of mNGS into diagnostic practice enables comprehensive pathogen detection from a single sample in complex CNS infections.

**Disclosures:**

Coren A. Milbury, PhD, Delve Bio: Employee|Delve Bio: Stocks/Bonds (Private Company) Brian O'Donovan, PhD, Delve Bio: Employee|Delve Bio: Stocks/Bonds (Private Company) Emily Crawford, PhD, Delve Bio: Inventor on provisional patent application|Delve Bio: Stocks/Bonds (Private Company) Nick Gimbrone, PhD, Delve Bio: Employee|Delve Bio: Stocks/Bonds (Private Company) Sirisha Thippabhotla, PhD, Delve Bio: Employee|Delve Bio: Stocks/Bonds (Private Company) Greg Gydush, BSc, Delve Bio: Current employee Fay Wang, PhD, Delve Bio: Employee|Delve Bio: Stocks/Bonds (Private Company) Anna Uehara, PhD, Delve Bio: Stocks/Bonds (Private Company) Timothy M. Blicharz, PhD, Delve Bio, Inc.: Employee|Delve Bio, Inc.: Stocks/Bonds (Private Company) Benisa Yuzawa, BBA, Delve Bio: Employee|Delve Bio: Stocks/Bonds (Private Company) Steve Miller, MD/PhD, Delve Bio: Employee|Delve Bio: Stocks/Bonds (Private Company)

